# Prevalence and predictors of delayed initiation of breastfeeding among postnatal women at a tertiary hospital in Eastern Uganda: a cross-sectional study

**DOI:** 10.1186/s13690-023-01079-2

**Published:** 2023-04-14

**Authors:** Loyce Kusasira, David Mukunya, Samuel Obakiro, Kiyimba Kenedy, Nekaka Rebecca, Lydia Ssenyonga, Mbwali Immaculate, Agnes Napyo

**Affiliations:** 1 Department of Nursing, Busitema University Faculty of Health Sciences, P.O. BOX 236, Mbale, Tororo, Uganda; 2 Department of Community and Public Health, Busitema University Faculty of Health Sciences, P.O. BOX 236, Mbale, Tororo, Uganda; 3 Department of Pharmacology and Therapeutics, Busitema University Faculty of Health Sciences, P.O. BOX 236, Mbale, Tororo, Uganda; 4 Sanyu Africa Research Institute, P.O BOX 2190, Mbale, Uganda; 5 Department of Public Health, Faculty of Health Sciences, Uganda Martyrs University, P.O. Box 5498, Kampala, Uganda

**Keywords:** Delayed initiation of breastfeeding, Early initiation of breastfeeding, Breastfeeding, Infants, Lactating women

## Abstract

**Background:**

The rates for the delayed initiation of breastfeeding in Uganda remain unacceptably high between 30% and 80%. The reasons for this are not well understood. We aimed to determine the prevalence and predictors for the delayed initiation of breastfeeding in Eastern Uganda.

**Methods:**

This study employed a cross-sectional study design. A total of 404 mother-infant pairs were enrolled onto the study between July and November, 2020 at Mbale regional referral hospital (MRRH). They were interviewed on socio-demographic related, infant-related, labour and delivery characteristics using a structured questionnaire. We estimated adjusted odds ratios using multivariable logistic regression models. All variables with p < 0.25 at the bivariate level were included in the initial model at the multivariate analysis. All variables with p < 0.1 and those of biological or epidemiologic plausibility (from previous studies) were included in the second model. The variables with odds ratios greater than 1 were considered as risk factors; otherwise they were protective against the delayed initiation of breastfeeding.

**Results:**

The rate of delayed initiation of breastfeeding was 70% (n = 283/404, 95% CI: 65.3 – 74.4%). The factors that were associated with delayed initiation of breastfeeding were maternal charateristics including: being single (AOR = 0.37; 95%CI: 0.19–0.74), receiving antenatal care for less than 3 times (AOR = 1.85, 95%CI: 1.07–3.19) undergoing a caesarean section (AOR = 2.07; 95%CI: 1.3–3.19) and having a difficult labour (AOR = 2.05; 95%CI: 1.25–3.35). Infant characteristics included: having a health issue at birth (AOR = 9.8; 95%CI: 2.94–32.98).

**Conclusions:**

The proportion of infants that do not achieve early initiation of breastfeeding in this setting remains high. Women at high risk of delaying the initiation of breastfeeding include those who: deliver by caesarean section, do not receive antenatal care and have labour difficulties. Infants at risk of not achieving early initiation of breastfeeding include those that have a health issue at birth. We recommend increased support for women who undergo caesarean section in the early initiation of breastfeeding. Breastfeeding support can be initiated in the recovery room after caesarean delivery or in the operating theatre. The importance of antenatal care attendance should be emphasized during health education classes. Infants with any form of health issue at birth should particularly be given attention to ensure breastfeeding is initiated early.

**Supplementary Information:**

The online version contains supplementary material available at 10.1186/s13690-023-01079-2.

## Background

The Uganda national policy guidelines on infant and young feeding recommend early initiation of breastfeeding (EIBF) within 1 h of birth, exclusive breastfeeding for 6 months and there after continued breastfeeding for 2 years and beyond while introducing nutritionally adequate and age appropriate complementary foods [1]. Colostrum, the yellowish sticky breast milk produced at the end of pregnancy and first days after birth is the perfect food for the new born and this can be tapped through EIBF [2]. Colostrum is a rich source of nutrients, contains protective factors with anti-infective action, immunoglobulin, cytokines, complement-system components, leukocytes, oligosaccharides, nucleotides, lipids, and hormones that interact with each other and with the mucous membranes of the digestive and upper respiratory tracts of infants, providing passive immunity as well as stimulation for the development and maturation of the infant’s immune system [3]. The antimicrobial factors present in colostrum and milk have some common characteristics, such as resistance to degradation by digestive enzymes, protection of the mucosal surfaces and elimination of bacteria without initiating inflammatory reactions [3]. Therefore infants who miss out on EIBF, will miss out on the colostrum and this puts them at risk of opportunistic infections like diarrhea as well as hospitalization [4, 5].

In some African and Asian settings, delayed initiation of breastfeeding has been associated with maternal-related characteristics which include: maternal education, mother being overweight, antenatal care attendance, maternal HIV infection, having a caesarean delivery and delivering out of a hospital setting. [6–11]. Available evidence has demonstrated that infants who start breastfeeding within 1 h of birth will most likely exclusively breastfeed [5, 12].

The Ugandan government through the ministry of health has put up initiatives to promote EIBF like provision of free antenatal care at public health facilities and health education on infant feeding during antenatal visits [1]. Frequent antenatal care promotes frequent interface with the healthcare system coupled with continuous health education which inevitably equips the mother with the required knowledge on the importance of EIBF and this definitely translates into practice. Despite these initiatives, the practice of EIBF in Uganda is divergent from the ideal practice that is recommended in the policy guidelines on infant and young child feeding [1, 12]. The rates of EIBF vary across and within countries [7, 8, 12–14] and Uganda is no exception [12, 14–16]. The reasons behind this discrepancy are not fully explored or understood and vary from context to context. There is also paucity of published evidence on this subject in Mbale city in Eastern Uganda. Given this background, it was vital for us to conduct this study to determine the prevalence and predictors for delayed initiation of breastfeeding in Eastern Uganda. The findings from this study will help in identifying groups of women and infants that are at risk of delaying the initiation to breastfeeding for their infants. These groups can then be a target for interventions of appropriate infant feeding counselling and support.

## Methods

### Study design and setting

This study employed a cross-sectional study design. Mother-infant pairs were enrolled onto the study between July and November, 2020 at Mbale Regional Referral Hospital (MRRH), found in Mbale city, Eastern Uganda. MRRH is a general and teaching hospital for several medical and nursing institutions. It has a 400-bed capacity and serves over ten neighbouring districts in Mbale sub-region as a referral hospital. These include Kibuku, Kween, Bududa, Busia, Kapchorwa, Budaka, Butaleja, Pallisa, Manafwa, Namisindwa, Butebo, Sironko, Bukwo, Bulambuli and Tororo. However, the hospital also receives some patients from as far as Teso and Karamoja sub-regions. MRRH offers a wide range of health care services and these include: surgery, psychiatry, paediatric care, ear-nose and throat, outpatient, eye care, laboratory and dental care. This clinic also hosts special clinics such as gynaecology and obstetrics which include antenatal, maternity, postnatal and infant / young child care services. All these services are at no cost to the patients. In the Ugandan health care setting, pregnant women are free to seek antenatal care, labour and delivery services at any public health facility of their choice. However, during routine antenatal care classes at public health facilities, these women are advised to seek these services at health facilities that are nearest to where they reside. When the woman goes into labour, she reports to a public health facility and is admitted at the maternity ward. After delivery in the labour suite, women and their infants are then transferred to the postnatal ward where they both are monitored continuously for any complications that could arise. While admitted in the postnatal ward, women have to move to the young child clinic to take their infants for immunization. It is also possible that women-infant pairs registered and delivered from other health facilities other than MRRH are referred to the postnatal clinic for observation and to the young child clinic for immunization or child care. This study was conducted at the postnatal and young child clinics to obtain information about the women and their infants.

### Participants and procedures

**Fig. 1:**
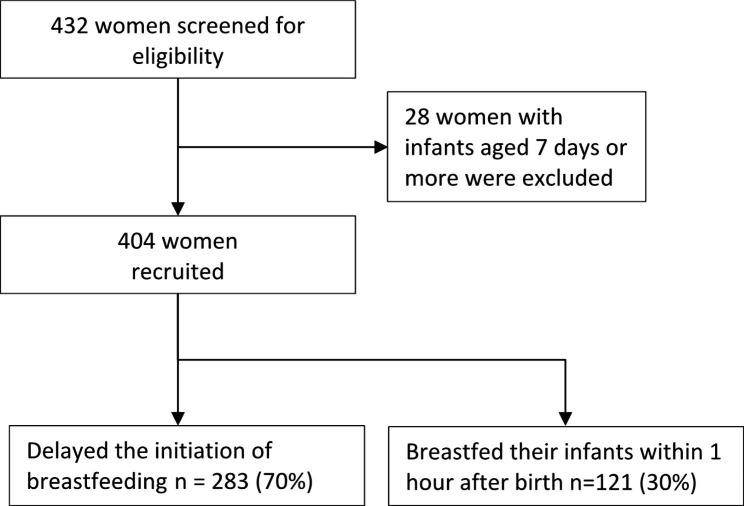
Study flow chart

We consecutively enrolled onto the study, mother-infant pairs who were receiving postnatal care and those attending the young child clinic at MRRH. We recruited women with infants who were aged 0–6 days. We excluded women with infants aged 7 days or more for the purpose of mitigating recall bias. After consenting, women were interviewed on socio-demographic-related, infant-related characteristics as well as issues surrounding their pregnancy, labour and delivery. The information was collected using questionnaires through face-to-face interviews by trained research assistants who were fluent in the local language. The questionnaires used during the interview process were translated from English to the local language. The data collection tool was pretested to remove any irrelevant questions and avoid misinterpretation. The research assistants were qualified midwives who had experience in conducting research and providing postnatal care as well as immunization. These research assistants were not employees of MRRH to overcome social desirability. While collecting data, research assistants had to implement COVID-19 preventive measures like use of face masks, wearing of plastic aprons, hand washing with soap and water. A total of 404 mother-infant pairs were included in the final analysis, Fig. 1.

### Study size

The sample size used in this study was determined using the Cochran formula stated as N= [Z^2^PQ]/d^2^ [17]. Where: N is the sample size, the Z- score at a 95% confidence level is 1.96. P, which is the prevalence of EIBF in Uganda, stands at 65% [16]. Q is (1-p). d is the acceptable margin of error which is 5% or 0.05. We assumed a non-response rate of 10%. The minimum sample size required for this study was 389, we however included 404 postnatal women and their infants.

### Variables

The outcome variable in our study was delayed initiation of breastfeeding defined as putting the infant on the breast to suckle beyond one hour after birth [1]. The exposures included in this study were maternal socio-demographic characteristics, pregnancy-, labour- and delivery- related as well as infant-related characteristics. Socio-demographic characteristics included maternal age which was recorded as the completed number of years for each individual; marital status was categorised and labelled as “married” if the women reported to be cohabiting or married. If she was divorced, separated or living alone, she was categorised as “single”. Religion was categorised as either “Christian” or “Moslem”. All those women that were formally or self-employed were categorised and labelled as “employed” and the rest as “unemployed”. Any residence that was estimated to be more than 10 km from Mbale municipality was categorised and labelled as “rural” or else “urban”. Women who had attended 3 or more antenatal visits were categorised and labelled as ‘≥ 3’ or otherwise they were labelled as ‘<3’. Women whose labour started between 06.00 and 18.59 h were categorised as “day-time onset of labour” and else “night-time onset of labour”. Women who delivered in any type of health care setting were all categorised as “hospital delivery” and otherwise as “non-hospital delivery”. Women who had reported to have had a lot of bleeding after delivery, prolonged labour, an episiotomy, high blood pressure, retained placenta, perineal tears, cervix not opening, breech presentation and draining liquor were all categorised and labelled as “had labour difficulty” or else “none”. Women who delivered their infants between 06.00 and 18.59 h were categorised and labelled as having delivered in the “day-time” and else “night-time”. An infant was categorised to have “had a health issue at birth” if the mother reported the infant to have weighed > 2.5 Kg, had difficulty in breathing, fever or diarrhoea. We, however, did not measure the body temperature of the infants due to the restrictive measures undertaken in MRRH for the prevention of SARS-CoV2. The age of the infants was recorded as completed number of days the infant had lived after birth.

### Data analysis and management

Data were entered into excel software by two independent entrants. The two databases were then merged to check for any inconsistencies data entry. The data was then exported for analysis into Stata version 14.0 (StataCorp, College Station, Texas, U.S.A.). Continuous data, if normally distributed, was summarised into means and standard deviations and if skewed, was summarised into medians with their corresponding interquartile ranges. Categorical variables were summarised into frequencies and percentages. The proportion of women who delayed to initiate breastfeeding was estimated and its confidence limits calculated using the exact method. We used multivariable generalized linear model regression analysis with a logit link to estimate the adjusted odds ratios of the independent variables on delayed initiation of breastfeeding while controlling for confounding. All variables with *p* < 0.25 at the bivariate level were included in the initial model at the multivariate analysis. All variables with *p* < 0.1 and those of biological or epidemiologic plausibility like age were included in the second model. We relied on the likelihood-ratio test to check the significant difference between the initial and the second model. There was no difference between the two models so then we adapted the second model. We checked for confounding by calculating the percentage change in each effect measure by removing or introducing one variable at a time into the second model. If a variable caused more than 10% change in any effect measure, then it was considered a confounder. We used the visual inspection factor to check for collinearity among all the variables that were included in the initial model. There was no collinearity. The pseudo R2 was 0.1146.

## Results

### Sociodemographic-related characteristics

The median age of women attending the postnatal clinic at MRRH was 24 years (IQR 20, 30). The majority of these women were married (87.1%, n = 221/404), Christian (66.8%), unemployed (75.5%) and lived in a rural residence (71.5%). Only 10% of the women had received a tertiary education. Most of the women (70%) had 3 or more antenatal visits while they were pregnant. About forty per cent (n = 156/404) had a difficult labour. More than half of the women delivered by caesarean Sect. (57.2%) and in a hospital setting (98.3%). Many of the infants born to these women had no health issue at birth (84.9%). Approximately fifty per cent of the infants born to these women were male (n = 206/404) and their mean weight was at birth 3.21 Kg (SD 0.62). The median age of these infants was 1 day (IQR 1,2). (Table 1).

### Prevalence of delayed initiation of breastfeeding

In our study, 70% (n = 283/404, 95% CI: 65.3 – 74.4%) of the postnatal women did not breastfeed their infants within one hour after birth (Table 1). We had a response rate of 100%. We achieved this by checking all questionnaires for completeness immediately after each interview prior to the participant leaving.

**Table 1:** Characteristics of mothers and their infants

	Initiation of breastfeeding			
Variable	Delayed N = 283 (70) N(%)	Early N = 121 (30) N(%)	Total (N) N = 404	P-value
**Mothers’ individual characteristics**				
**Age (years)**				
15–20	55(19.4)	26(21.5)	81 (20.0)	0.766
20–29	154(54.4)	67(54.4)	221 (54.7)	
30–47	74(26.2)	28(23.1)	102(25.3)	0.599
**Marital status**				
Married	253(89.4)	99(81.8)	352(87.1)	
Single	30 (10.6)	22(18.2)	52(12.9)	0.039*
**Religion**				
Christian	195(68.9)	75(62.0)	270(66.8)	
Moslem	88(31.1)	46(38.0**)**	134(33.2)	0.177*
**Education level**				
Primary	134(47.3)	48(39.7)	182(45.0)	
Secondary	121(42.8)	61(50.4)	182(45.0)	0.138*
Tertiary	28(9.9)	12(9.9)	40(10.0)	0.64
**Employment status**				
Not employed	219(77.4)	86(71.1)	305(75.5)	
Employed	64(22.6)	35(28.9)	99(24.5)	0.178*
**Residence**				
Urban	72(25.4)	43(35.5)	115(28.5)	
Rural	211(74.6)	78(64.5)	289(71.5)	0.04*
**Characteristics during pregnancy, labour and delivery**				
**Number of ANC visits**				
≥3	191(67.5)	94(77.7)	285(70.5)	
<3	92(32.5)	27(22.3)	119(29.5)	0.041*
**Labour onset**				
Day-time	126(44.5)	59(48.8)	185(45.8)	
Night-time	130(45.9)	51(42.2)	181(44.8)	0.439
No pains	27(9.6)	11(9.0)	38(9.4)	0.722
**Attendant in labour**				
Husband	58(20.5)	23(19.0)	81(20.1)	
Other	225(79.5)	98(81.0)	323(79.9)	0.733
**Labour difficulties**				
None	161(56.9)	87(71.9)	248(61.4)	
Had labour difficulty	122(43.1)	34(28.1)	156(38.6)	0.005*
**Mode of delivery**				
Normal delivery	105(37.1)	68(56.2)	173(42.8)	
Caesarean section	178(62.9)	53(43.8)	231(57.2)	0.000*
**Time of delivery**				
Day time	162(57.2)	62(51.2)	224(55.5)	
Night time	121(42.8)	59(48.8)	180(44.5)	0.267
**Place of delivery**				
Hospital	277(97.9)	120(99.2)	397(98.3)	
Non-hospital	6(2.1)	1(0.8)	7(1.7)	0.379
**Characteristics of the infants**				
**Infant’s gender**				
Male	146(51.6)	60(49.6)	206(51.0)	
Female	137(48.4)	61(50.4)	198(49.0)	0.712
**Infant’s weight**				
Less than 2.5 kg	29(10.3)	5(4.1)	34(8.4)	0.049*
2.5 to 4.5 kg	248(87.6)	114(94.2)	362(89.6)	
Greater than 4.5 kg	6(2.1)	2(1.7)	8(2.0)	0.697
**Infant’s age**				
Less than 3 days	205(72.4)	104(85.9)	309(76.5)	
Greater or equal to three days	78(27.6)	17(14.1)	95(23.5)	0.04*
**Birth order**				
1	102(36.0)	46(36.0)	148(36.6)	
2	59(20.9)	24(19.8)	83(20.5)	0.731
3	122(43.1)	51(42.2)	173(42.9)	0.755
**Infant had a health issue at birth**				
No	225(79.5)	118(97.5)	343(84.9)	
Yes	58(20.5)	3(2.5)	61(15.1)	< 0.001*

*Variables that were included in the initial multivariable model during multivariable analysis.

### Predictors for delayed initiation of breastfeeding

Women who were single were less likely to delay the initiation of breastfeeding (AOR = 0.37; 95%CI: 0.19–0.74) compared to those who are married. Women who had received antenatal care less than 3 times were more likely to delay the initiation of breastfeeding (AOR = 1.85, 95%CI: 1.07–3.19) compared to those that had received antenatal care 3 or more times. Women who underwent a caesarean delivery were more likely to delay the initiation of breastfeeding (AOR = 2.07; 95%CI: 1.30–3.19) compared to women who had a spontaneous vaginal delivery. Women who had a difficult labour were more likely to delay the initiation of breastfeeding (AOR = 2.05; 95%CI: 1.25–3.35) compared to those that did not have any difficulty during labour. Infants that had a health issue at birth were less likely to start breastfeeding within 1 h after birth (AOR = 9.80; 95%CI: 2.94–32.98) when compared with those that did not have a health problem (Table 2).

**Table 2:** Factors influencing early initiation of breastfeeding

Variable	Unadjusted OR	Adjusted OR		
**Age (years)**				
15–20	0.92 (0.53–1.59)	0.84 (0.45–1.57)		
20–29	1		1	
30–47	1.15 (0.68–1.94)	1.07 (0.61–1.87)		
**Marital status**				
Married	1	1		
Single	0.53 (0.29–0.97)	0.37 (0.19–0.74)*		
**Baby had a health issue at birth**				
No	1	1		
Yes	10.14 (3.11–33.05)	9.80 (2.94–32.98)*		
**Number of ANC visits**				
≥3	1	1		
<3	1.68 (1.02–2.75)	1.85 (1.07–3.19)*		
**Mode of delivery**				
Normal delivery	1	1		
Caesarean section	2.18 (1.41–3.35)	2.07 (1.30–3.29)*		
**Labour difficulties**				
None	1	1		
Had labour difficulty	1.94 (1.22–3.07)	2.05 (1.25–3.35)*		

*significant predictors for the delayed initiation of breastfeeding

## Discussion

In this study we found that an unacceptably high proportion of women delayed the initiation of breastfeeding after birth. Studies done between 2013 and 2021 in African countries including Uganda, Nigeria, Ghana, Zimbabwe, South Sudan, Ethiopia and Tanzania demonstrates that the rates of EIBF vary between and within countries ranging from 30 to 83% [7–10, 12–15, 18–20]. Reasons for these findings also vary across contexts. Several studies explain that caesarean delivery is associated with delayed initiation of breastfeeding [7–10, 13, 14, 18–23]. In our cohort, more than half of the women underwent caesarean section delivery and this could possibly explain the high rate of delayed initiation of breastfeeding. A considerable number of these women in this study also had some form of labour difficulty which was most likely an indication for the caesarean section. A number of infants born to these women also had a health issue after birth probably due to a difficult labour. These infants are likely to have difficulty in sucking. The combination of a caesarean delivery and the baby having a health issue at birth may further contribute to the risk of late breastfeeding initiation. Therefore, mothers who had undergone caesarean section may be less likely to introduce their new born infants to breastfeeding within the recommended one hour after birth.

Consistent with studies done elsewhere [7–10, 13, 14, 18–23], our study found that delivery by caesarean section increased the odds of delayed initiation of breastfeeding. Along with caesarean delivery, comes exhaustion arising from the procedure itself and the effects of anaesthesia which may impede the early initiation of breastfeeding. A caesarean section birth takes a lot of time involving the repair of surgical incisions and recovery which may contribute to late breastfeeding initiation.

We found that infants that had a health issue at birth were more likely to delay to start breastfeeding after birth. This finding is consistent with evidence found in other studies [24, 25]. These health issues included difficulty in breathing, fever, diarrhoea and the infant being too weak. Infants with health issues at birth may cause the infant to have difficulty in suckling due to weak breastfeeding reflexes, poor coordination and lack of ability to swallow. Further explanations to this effect have already been tucked in the earlier paragraphs.

Women who had received antenatal care less than 3 times while they were pregnant were less likely to initiate breastfeeding within 1 h after birth. This association has been demonstrated in other studies [8, 12, 22, 24, 26]. During antenatal care, the benefits of EIBF are always emphasized in health education talks in MRRH. The more the antenatal care visits the women have, the more the interface they make with these health education talks. In this way, these women become more conversant with these counselling messages and are therefore more likely to support their infants in initiating breastfeeding within 1 h after birth.

Women who had a difficult labour were more likely to delay the initiation of breastfeeding. This finding is consistent with evidence found elsewhere [8, 23, 27]. Difficult labour in our study included prolonged labour, body weakness, experiencing a lot of pain, prolonged bleeding and having received an episiotomy. Most of the women that had a difficult labour actually ended up giving birth by caesarean section. This mode of delivery could have contributed to the delay in EIBF as explained in earlier paragraphs. In addition, maternal and foetal indications for caesarean delivery and postoperative care disrupt bonding and mother-infant interaction and delay initiation of breastfeeding.

In our study, women who were single were more likely to practice EIBF. Other studies [13, 24] have found contrary evidence to ours. We recommend that a qualitative study can be conducted on this subject matter to best understand the occurrence of this association in our context.

### Strengths and limitations

This study was done in a regional referral hospital. Women who deliver at this hospital are probably referred from other lower cadre health facilities due to complications in pregnancy and may most probably require more specialized clinical care. We therefore could have overestimated the prevalence of EIBF. Our study findings may only be generalizable to this nature of population or those similar to it. We never asked any questions on cultural practices that may influence EIBF or why the infants were initiated late. We neither investigated any health system-related factors nor maternal knowledge on EIBF and how these could influence our outcome of interest. We relied on self-reporting of the mother to report on the time of delivery (for both those who delivered normally and by caesarean section) and time of initiation of breastfeeding from which we computed the time interval. This study had some strength, too. We conducted this study among infants that did not exceed 6 days of age. This recall period could have helped to counteract the possibility of recall bias.

## Conclusions and recommendations

The proportion of infants that do not achieve EIBF in this setting remains unacceptably high at 70%. Women at high risk of delaying the initiation of breastfeeding include those who: deliver by caesarean section, do not receive antenatal care and have labour difficulties. Infants at risk of not achieving EIBF include those that have a health issue at birth. We recommend increased support in the early initiation of breastfeeding for women who undergo caesarean section by introducing baby-friendly initiatives in hospitals like initiating breastfeeding support in the recovery room after caesarean delivery or in the operating theatre. The importance of antenatal care attendance should be emphasized during health education classes and any other community / public forums like media. Infants with any form of health issue at birth should particularly be given attention to ensure breastfeeding is initiated early. We also recommend a qualitative investigation into the reasons as to why women in this setting delay to initiate breastfeeding for their newly born infants.

## Electronic supplementary material

Below is the link to the electronic supplementary material.

Supplementary Material 1

## Data Availability

The datasets used and/or analysed during the current study are available from the corresponding author on reasonable request.

## Abbreviations

ANC: Antenatal care
AOR: Adjusted odds ratio
CI: Confidence interval
COVID-19: Coronavirus disease of 2019
EIBF: Early initiation of breastfeeding
MRRH: Mbale regional referral hospital
SARS-CoV2: Severe acute respiratory syndrome coronavirus 2
