# Supplementary material for: Prevalence and predictors of delayed initiation of breastfeeding among postnatal women at a tertiary hospital in Eastern Uganda: a cross-sectional study
Source: Arch Public Health. 2023 Apr 14;81:56. doi: 10.1186/s13690-023-01079-2 (PMC10102688; doi:10.1186/s13690-023-01079-2)
Supplement: Supplementary file 1 — Supplementary Material 1 [file 13690_2023_1079_MOESM1_ESM.pdf]

Agnes Napyo
Busitema University Faculty of Health Sciences
P.O. BOX 236, Tororo
Uganda

1/12/2022

To the Editor-in-Chief,
Archives in Public Health Journal

Dear Editor,

Thank you for giving us the opportunity to submit our paper entitled, "Prevalence and predictors of delayed initiation of breastfeeding among postnatal women at a tertiary hospital in Eastern Uganda: a cross-sectional study" to the Archives of Public Health journal. As stipulated in the guidelines for submission under step 1: Aims and Scope, it is indicated that authors who want to submit a manuscript to Archives of Public Health for publication should provide a statement to the Editors as addendum to the letter to the Editor.
As such, we have revised in accordance to your comments and have come up with a response to each point that you raised. The details are as follows:

What is known?
Colostrum, the yellowish sticky breast milk produced at the end of pregnancy and first days after birth is the perfect food for the new born and this can be tapped through EIBF. Colostrum is a rich source of nutrients, contains protective factors with anti-infective action, immunoglobulin, cytokines, complement-system components, leukocytes, oligosaccharides, nucleotides, lipids, and hormones that interact with each other and with the mucous membranes of the digestive and upper respiratory tracts of infants, providing passive immunity as well as stimulation for the development and maturation of the infant's immune system. The antimicrobial factors present in colostrum and milk have some common characteristics, such as resistance to degradation by digestive enzymes, protection of the mucosal surfaces and elimination of bacteria without initiating inflammatory reactions. Therefore infants who miss out on EIBF, will miss out on the colostrum and this puts them at risk of opportunistic infections like diarrhea as well as hospitalization. The Uganda national policy guidelines on infant and young feeding recommend early initiation of breastfeeding (EIBF) within 1 hour of birth, exclusive breastfeeding for 6 months and there after continued breastfeeding for 2 years and beyond while introducing nutritionally adequate and age appropriate complementary foods. In some African and Asian settings, delayed initiation of breastfeeding has been associated with maternal-related characteristics which include: maternal education, mother being overweight, antenatal care attendance, maternal HIV infection, having a caesarean delivery and delivering out of a hospital setting. . Available, evidence has demonstrated that infants who start breastfeeding within 1 hour of birth will

most likely exclusively breastfeed

What does the study adds?
Despite these initiatives, the practice of EIBF in Uganda is divergent from the ideal practice that is recommended in the policy guidelines on infant and young child feeding. The rates of EIBF vary across and within countries and Uganda is no exception. The reasons behind this discrepancy are not fully explored or understood and vary from context to context. There is also paucity of published evidence on this subject in Mbale city in Eastern Uganda. Given this background, it was vital for us to conduct this study to determine the prevalence and predictors for delayed initiation of breastfeeding in Eastern Uganda.

What are implications for clinical practice, public health and / or research?
The findings from this study will help in identifying groups of women and infants that are at risk of delaying the initiation to breastfeeding for their infants. These groups can then be a target for interventions of appropriate infant feeding counselling and support.

Yours sincerely,

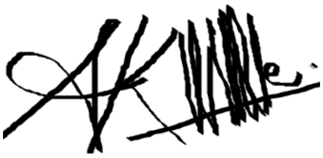

Dr. Napyo Agnes Kasede
Corresponding author
